# An update on renal tubular injury as related to glycolipid metabolism in diabetic kidney disease

**DOI:** 10.3389/fphar.2025.1559026

**Published:** 2025-04-24

**Authors:** Anqi Feng, Ruili Yin, Rong Xu, Baoyu Zhang, Longyan Yang

**Affiliations:** ^1^ Center for Endocrine Metabolic and Immune Diseases, Beijing Luhe Hospital, Capital Medical University, Beijing, China; ^2^ Beijing Key Laboratory of Diabetes Prevention and Research Care, Beijing Luhe Hospital, Capital Medical University, Beijing, China

**Keywords:** diabetic kidney disease, renal tubular epithelial cells, glucose metabolism, lipid metabolism, therapeutic medications

## Abstract

Diabetic kidney disease (DKD) is a severe microvascular complication of diabetes, which can result in end-stage renal disease (ESRD). As the main site of renal reabsorption and its exposed environment, renal tubules can be damaged by various factors. Recent studies have shown that renal tubular epithelial cells (RTECs) injury plays an important role in the occurrence and progression of DKD. The glycolipid metabolism disorders are a vital factor contributing to RTECs injury, which in turn affects the progression of DKD. Abnormal glucose and lipid metabolism can cause oxidative stress, mitochondrial damage, cell apoptosis and lipid accumulation, which can cause RTECs injury. Therefore, this review describes the main pathological mechanism of the injury caused by glycolipid metabolism and the corresponding therapeutic drugs in the clinical treatment of DKD.

## Highlights

• The disorders of glycolipid metabolism play an important role in the injury of renal tubular epithelial cells (RTECs), leading to the occurrence and development of diabetic kidney disease (DKD).
• This review summarizes the molecular mechanisms of the damage of RTECs caused by glycolipid metabolism, as well as corresponding therapeutic drugs for prevention and treatment of DKD.

## 1 Introduction

Diabetic kidney disease (DKD) is one of the microvascular complications of diabetes. It is characterized by persistent proteinuria, decreased glomerular filtration rate (GFR), and characteristic pathological changes (such as glomerular basement membrane thickening and mesangial dilation), and it can eventually progress to end-stage renal disease (ESRD) (Navaneethan et al., 2023; ElSayed et al., 2023). It is characterized by a gradual deterioration in renal function and recurrent albuminuria (Chen et al., 2022a; Han et al., 2017). DKD is the primary cause of kidney failure globally, affecting 25%–40% of persons with diabetes mellitus DM (Jung and Yoo, 2022). Renal vascular disease, tubulointerstitial fibrosis, and glomerulosclerosis are pathological alterations in DKD (Gupta et al., 2023). About a third of individuals with type 1 diabetes (T1DM) and almost half of those with type 2 diabetes (T2DM) may experience chronic kidney disease (CKD), which is typified by either increased urine albumin excretion or reduced renal function (Thomas et al., 2015). When albuminuria is discovered, the lesions are frequently at an advanced stage, resulting in a rapid loss in kidney function toward end-stage renal disease (ESRD).

**FIGURE 1:**
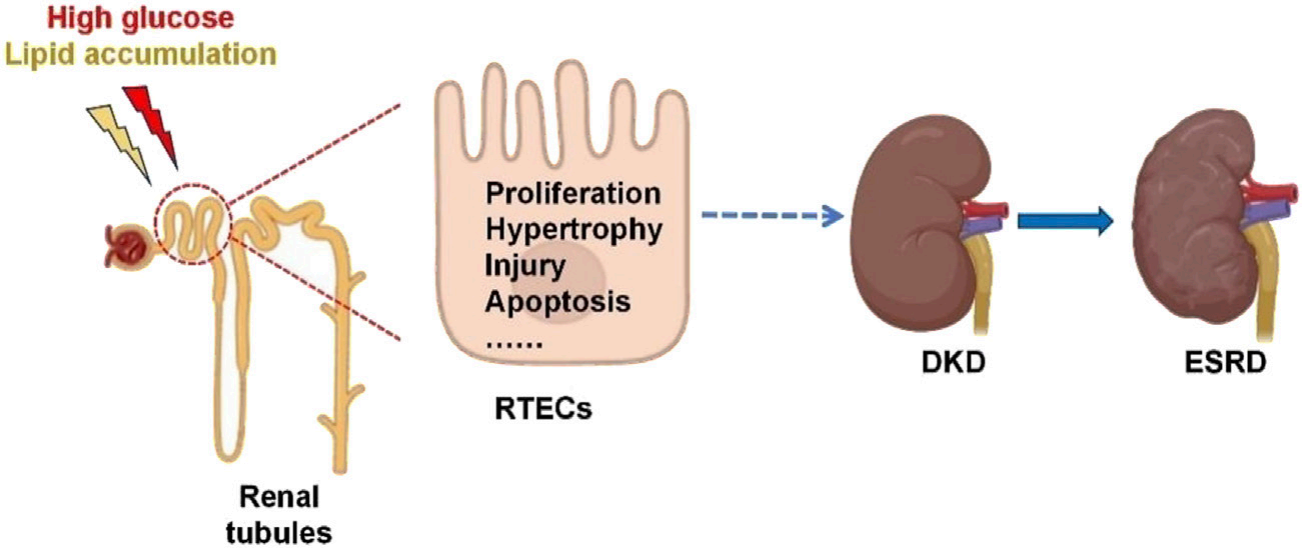
Renal tubular injury in the development of diabetic kidney disease. Glucolipid imbalance can trigger renal tubulointerstitial fibrosis, hypertrophy, injury and apoptosis, leading to DKD and eventually ESRD. RTECs, renal tubular epithelial cells; DKD, diabetic kidney disease; ESRD, end-stage renal disease.

Renal tubules are the main site of renal reabsorption and composed of the proximal tubule, Henle’s loop, the distal tubule and the collecting duct (Zhou X. et al., 2023). Renal tubular epithelial cells (RTECs), which constitute the predominant cell type within the renal tubules, are highly vulnerable to metabolic dysregulation and inflammatory conditions. These pathological changes can subsequently trigger fibrotic processes and contribute to systemic inflammation (Chen et al., 2020). A previous study has revealed that RTECs are closely associated with worsening renal function in DKD, and that tubular injury is present in the initial phases of DKD (Ruilope et al., 2019). Tubular injury refers to the structural and functional abnormalities of RTECs caused by ischemia, toxins, inflammation or metabolic disorders, which are manifested as dysfunction of reabsorption, secretion and concentration (ElSayed et al., 2023). In DKD, renal tubular injury is characterized by several pathological features, including thickening of the tubular basement membrane, inflammatory lesions within the tubules, tubular atrophy, elevated apoptotic activity, interstitial fibrosis, and rarefaction of peritubular capillaries (Chen et al., 2020; Liu et al., 2019). Abnormal glycolipid metabolism refers to the coexistence of glucose metabolism disorders (such as hyperglycemia and insulin resistance) and lipid metabolism disorders (such as hypertriglyceridemia, etc.), commonly seen in metabolic syndrome, obesity, and T2DM (Tian et al., 2023). Dysregulated glucose and lipid metabolism induces oxidative stress and inflammatory cascades within renal tubules, which subsequently drive interstitial fibrosis and tubular atrophy (Jung and Yoo, 2022). These pathological changes contribute to the progression of diabetic kidney disease (DKD) and ultimately culminate in end-stage renal disease (ESRD). A schematic representation of this mechanistic pathway is provided in Figure 1.

Current research prioritizes key signaling pathways regulating lipid metabolism and inflammation to identify therapeutic targets, alongside emerging intervention strategies. This review comprehensively examines the molecular mechanisms underlying glycolipid dysregulation in RTECs and its pivotal role in the pathogenesis of DKD. We further summarize current pharmacological agents targeting these pathways, either directly or indirectly, to inform precise therapeutic strategies for DKD. The following section highlights recent advances in understanding glycolipid metabolism disorders in renal tubular injury and their implications for DKD prevention and treatment.

## 2 Method

The keywords of the review include: “glucolipid metabolism,” “diabetic nephropathy,” “renal tubule injury,” “renal tubular epithelial cell,” “hypoglycemic drugs,” “lipid-lowering drugs” and “traditional Chinese medicine.” The search was conducted using multiple online databases, including PubMed, Web of Science and ScienceDirect, to retrieve English-language journal reports and articles published up to December 2024. Manually filter references from extracted articles.

## 3 RTECs injury

Renal tubules and tubulointerstitial tissue make up over 90% of the renal parenchyma. The renal tubules’ primary role is reabsorption, which exposes them to glomerular filtrate, peritubular capillary blood supply, and tubulointerstitial environment, making them more vulnerable to injury by numerous proinflammatory and profibrotic chemicals (Thomas et al., 2015). Tubular cell hypertrophy, tubular basement cell membrane thickening, and interstitial inflammation with monocyte infiltration are the primary pathophysiological alterations in early DKD.

**FIGURE 2:**
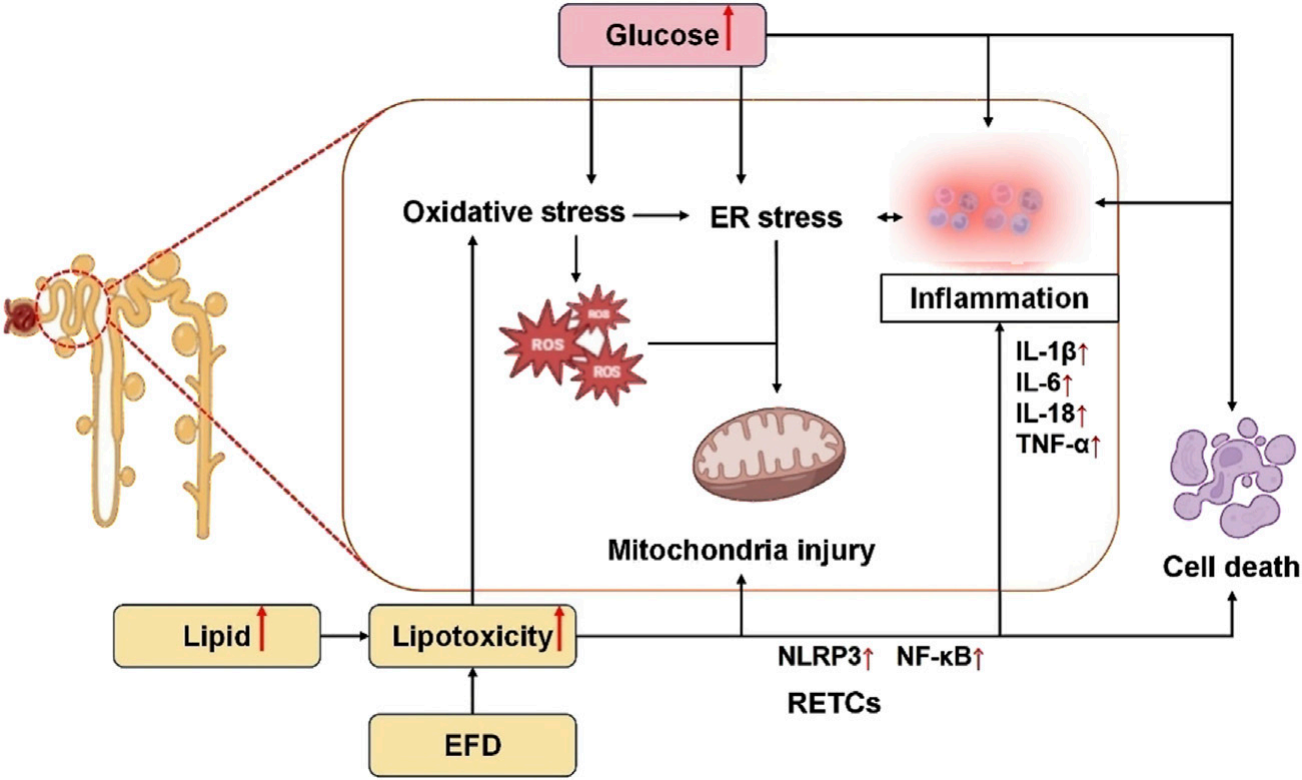
Mechanisms of RTECs injury by disorders of glycolipid metabolism. Elevated glucose levels induce oxidative stress, endoplasmic reticulum (ER) stress, inflammatory activation, and cellular apoptosis. Specifically, oxidative stress promotes ROS generation, which synergizes with ER stress to disrupt mitochondrial integrity, thereby exacerbating RTEC injury. Concurrently, hyperlipidemia and excessive EFD exacerbate lipid toxicity, directly triggering oxidative stress, mitochondrial dysfunction, and inflammatory cascades, ultimately leading to RTEC apoptosis and tubular damage. EFD, ectopic fat deposition; ER, endoplasmic reticulum; ROS, reactive oxygen species; RTECs, renal tubular epithelial cells; NLRP3, nucleotide-binding oligomerization domain-like receptor 3; NF-κB, nuclear factor κB; IL-1β, interleukin-1beta; IL-6, interleukin-6; IL-18, interleukin-18; TNF-α, tumor necrosis factor α.

DKD is a multifaceted disease with many pathways, yet the bulk of its causes are related to issues with lipid and glucose metabolism (Gillard et al., 2020; Gewin, 2021). The kidneys, recognized as highly energy-demanding organs, derive their substantial metabolic energy primarily through mitochondrial oxidative phosphorylation of glucose and fatty acids to sustain their physiological functions (Pan, 2022). Glucose and lipid metabolism are intimately linked processes. Glucose and lipids provide the main energy and are involved in all aspects of cellular metabolism. Disruptions in glycolipid metabolism can cause cellular oxidative stress, apoptosis, and other negative effects in the DKD population (Chen et al., 2020). Disruptions in glycolipid metabolism can cause mitochondrial malfunction, reactive oxygen species (ROS), inflammation, and abnormal secretion of adipokines (Wang et al., 2023). Abnormal glucose and lipid metabolism mainly promotes renal tubular cell injury through the following aspects, which are summarized in Figure 2.

### 3.1 Lipid accumulation, adipokines and ectopic fat deposition (EFD)

It has been discovered that the kidney injury molecule (KIM-1), a protein belonging to the immunoglobulin class, accelerates the course of diabetic kidney disease by modulating renal tubular cells’ absorption of fatty acids (Kammer et al., 2019). Mori et al. stated that KIM-1 facilitates the proximal tubular absorption of palmitic acid-bound albumin, which worsens tubule damage by causing interstitial inflammation and fibrosis as well as secondary glomerulosclerosis in DKD (Mori et al., 2021). The KIM-1 tiny-molecule blocker TW37 is also mentioned as a possible therapy for the wound (Mori et al., 2021). Besides the above, hypercholesterolemia also causes lipid accumulation in kidney tissue, reducing oxygen transport by diffusion even further in early studies (Kammer et al., 2019; Song Y. et al., 2021). Lipid accumulation is a representative quality of tubular cells in DKD. Poor eating habits like overindulging in hypercaloric diets with high fructose and fat content are linked to the emergence of metabolic abnormalities like obesity, diabetes, and dyslipidemia. Diets high in saturated fat result in lower basal metabolic rates than diets high in unsaturated fatty acids (Luukkonen et al., 2018). Oil Red O staining revealed that the proximal tubule was the main location of lipid buildup, a finding that was also seen in the kidney of DKD patients and mice model (Yang et al., 2018). Damage to the glomeruli and tubule-interstitial tissues is caused by structural and functional alterations brought on by tissue lipotoxicity (Meléndez-Salcido et al., 2022).

Many adipocyte-derived proteins play an important role in DKD. Different adipokines play different regulatory roles. Leptin increased the expression of insulin-induced gene 1 (Insig-1) by activating adenosine 5′-monophosphate (AMP)-activated protein kinase (AMPK) to reduce EFD in palmitic acid-induced RTECs (Song A. et al., 2021). AdipoR1 expression was much lower in DKD patients and associated with both autophagosome assembly and renal function. AdipoRon contributes to the increased lipid accumulation and altered lipophagy in HK-2 cells exposed to the HG environment (Han et al., 2021). Partially counteracting the effects of AdipoRon on HK-2 cell lipophagy enhancement, EFD reduction, and fibrosis was AdipoR1 siRNA (Small Interfering Ribonucleic Acid) and UNC-51 like autophagy activating kinase 1 inhibitor (Jaishy and Abel, 2016).

However, some adipokines are increased in DKD. For instance, aspirin was a recently discovered hormone that might stimulate hepatic glucose release. T2DM patients have higher circulating asprosin stages, which are associated with preliminary diabetic kidney impairment (Zhang et al., 2020). Visfatin promotes cholesterol buildup in macrophages by increasing the storage of CD36 and scavenger receptor-A (Zhou et al., 2013).

Recently, the term “EFD” was coined to describe the lipid deposition in non-adipose tissues that occurred in a diabetic rat model. When a high-fat diet (HFD) induces eating pressure, adipose tissue’s surplus lipids are discharged into the bloodstream and accumulate in other organs such as the kidney, pancreas, liver, and blood vessels (Maharjan et al., 2021). The HFD triggers the pro-apoptotic pathway and causes increased oxidative stress, fat accumulation, and diminished mitochondrial function (Sun et al., 2020). Therefore, EFD-related organ dysfunction is significantly influenced by HFD-induced dyslipidemia. DKD patients’ tubular cells showed decreased lipophagy, increased EFD, and lipotoxicity, as well as decreased expression of AdipoR1. AdipoRon, an adiponectin receptor activator that stimulates autophagy, was found to correct similar alterations in db/db mice (Han et al., 2021). Thus, lipophagy deficit was linked to renal damage and EFD in DKD patients. Miguel et al. identified fatty acid oxidation (FAO) in RTECs as a promising therapeutic target for treating renal fibrosis in a recent investigation (Miguel et al., 2021). Furthermore, elevated expression of sterol regulatory element–binding protein-1 is caused by the high concentration of triglycerides (TG) and cholesterol (CHOL) in the renal tubules, which ultimately results in renal failure. Increased fat production and ingestion decrease β-oxidation, and cholesterol export can all impact lipid levels.

The term “lipotoxicity” refers to the abnormal regulation of intracellular components and the lipid environment, which leads to the accumulation of toxic lipids, abnormal activation of intracellular signaling pathways, chronic inflammation, and the death of RTECs (Ren et al., 2023).

### 3.2 Oxidative stress and mitochondrial injury

Oxidative overload has played an essential part in the development of renal damage in patients with diabetes (Wei et al., 2018). Oxidative stress is the consequence of pro-oxidant enzymes producing more reactive oxygen species (ROS) and antioxidants being depleted at the same time. The unifying mechanism connecting altered metabolic pathways in the kidneys with aberrant renal hemodynamics, which is known to be associated with DKD, has been identified as the overproduction of ROS (Jha et al., 2016).

Hyperglycemia is a major cellular stressor in the kidney, alters cellular metabolism in endothelial cells and podocytes, imposes an excessive workload on proximal tubule cells, and leads to early adaptive cellular hypertrophy and actin cytoskeleton remodeling (Mohandes et al., 2023). Renal ROS generation in diabetes is primarily mediated by various nicotinamide adenine dinucleotide phosphate (NADPH) oxidases (NOXs), but a faulty antioxidant system and mitochondrial dysfunction may also play some part (Jha et al., 2016). Malondialdehyde (MDA) level and superoxide dismutase (SOD, an antioxidant enzyme) activity are routinely utilized oxidative stress indicators. Compared with the normal group, SOD activity decreased and MDA levels increased in streptozotocin (STZ) induced diabetic rats (Wang X. et al., 2017), and levels of glutathione (GSH, a non-enzymatic antioxidant) are similarly lowered in the kidneys of rats with DKD (Antar et al., 2022). Additionally, an *in vitro* study indicated that in high glucose (HG) induced human proximal tubular (HK-2) cells, HG can increase both cellular and mitochondrial ROS (Lee et al., 2019). The formation of ROS within the mitochondria will cause damage to mitochondrial deoxyribonucleic acid (mtDNA), respiratory chain enzymes, and mitochondria dysfunction (Hu et al., 2023).

HG also induces defective mitochondrial autophagy, mitochondrial malfunction and apoptosis in HK-2 cells (Xiao et al., 2017). Given that the kidneys are the body’s second-most oxygen-consuming organ, they are significantly sensitive to mitochondrial dysfunction (Forbes and Thorburn, 2018). Mitochondrial abnormalities increase oxidative stress and activate inflammatory pathways, resulting in a gradual decrease in kidney function and fibrosis (Mohandes et al., 2023). Jiang et al. showed that compared to diabetics without renal impairment, DKD patients had lower mtDNA copy numbers and more mtDNA damage (Jiang et al., 2019). Mitochondrial fragmentation was particularly seen in renal tubules. Renal tubules and peripheral blood mononuclear cells (PBMCs) experienced an increase in ROS generation, the beginning of programmed cell death and a reduction in the potential of the mitochondrial membrane brought on by the build-up of damaged mtDNA and broken mitochondria (Jiang et al., 2019). Li et al. concluded that renal tubular cells of db/db mice have aberrant mitochondria, including impaired mitochondrial autophagy, overexpressed mitochondrial ROS, and fragmented mitochondria (Xiao et al., 2017). At present, there are many therapeutic drugs for mitochondria, including Finerenone, sodium glucose co-transporter 2 inhibitors, traditional Chinese medicine such as Huangkui capsule and so on (Yao et al., 2023; Peng et al., 2022; Zhu et al., 2023). Numerous investigations suggest that diabetes causes direct damage to renal tubules, resulting in mitochondrial dysfunction, such as decreased bioenergetics, overproduction of mitochondrial ROS, defective mitophagy, and dynamics disturbances, which trigger a series of metabolic abnormalities (Liu et al., 2020; Liu et al., 2024a; Zhou Y. et al., 2023; Yao et al., 2022). Understanding the pathobiology of tubulointerstitial cells would help researchers find novel biomarkers for DKD (Yao et al., 2022).

The energy source of renal mitochondria is mostly derived from the conversion of fatty acids, which includes the Krebs cycle and fatty acid oxidation through regulated enzyme systems. Mitochondria in the kidney use a variety of metabolic routes, including peroxisome proliferators-activated receptors (PPARs), cluster of differentiation 36 (CD36) signaling, and adenosine 5′-monophosphate (AMP)-activated protein kinase/peroxisome proliferator-activated receptor-γ coactivator-1 (AMPK/PGC-1), to preserve energy balance for both dynamic and static requirements (Han et al., 2021). The Krebs cycle’s substrates in the proximal tubules are amino acids and lipids, which produce a substantial amount of ATP. In the early stages of diabetes, the Krebs cycle is boosted in response to hyperfiltration but diminished cell performance in the later stages (Hasegawa and Inagi, 2020). The overconsumption of fatty acids can lead to incomplete oxidation in mitochondria, which generates ROS and oxidative stress, both of which can lead to mitochondrial dysfunction. Additionally, ROS may facilitate RTECs apoptosis (Sakashita et al., 2021). Apart from generating energy and ROS, mitochondria are essential for numerous cellular processes such as differentiation, apoptosis, necrosis, inflammation, and adaptability. By directly or indirectly influencing the stability or activity of different metabolic enzymes, mitochondrial reactive oxygen species (mtROS), also function as second messengers to control metabolic pathways (Liu et al., 2024b; Shi and Dansen, 2020). An *in vitro* investigation showed that increased glucose led to increased production of cytosolic phospholipase A2 (cPLA2), which in turn caused altered lipid metabolism in HK-2 cells, including the creation of lipid droplet and the accumulation of sphingosine-1-phosphate (Hou et al., 2024). Among all these studies suggested that RTECs have been found to undergo alterations in their condition due to diabetes-induced metabolic abnormalities, including the reprogramming of lipid metabolism. Of all the metabolic perturbations, mitochondria are central regulators. One of the main causes of diabetic tubular injury is thought to be mitochondrial dysfunction, which is correlated with an increase in mtROS generation. Therefore, the irreplaceable role of RTECs injury in diabetic kidney disease can also be reflected.

Homeostasis in the mitochondria, ER, and lysosomes depends on the multifunctional sorting protein known as phosphoric acidic cluster sorting protein 2 (PACS-2) (Li et al., 2020). PACS-2 reduces RTECs injury by promoting ER-mitochondria contact and mitophagy. PACS-2 expression has a negative correlation with the severity of tubulointerstitial diseases and a positive correlation with renal function (Li C. et al., 2022). Thus, in patients with diabetes, lipid-related renal impairment is made worse by PACS-2 deficiency in RTECs (Zhao et al., 2022).

### 3.3 Endoplasmic reticulum (ER) stress

A growing body of research suggests that renal disease development and progression are facilitated by the disruption of ER homeostasis (Yao et al., 2022; Wu et al., 2023). An imbalance in the cellular endoplasmic reticulum’s normal function and equilibrium is known as ER stress (Wang et al., 2023); it is closely connected with renal tubular dysfunctions in DKD patients. In renal tubules with DKD, impaired protein N-glycosylation causes ER stress, which in turn sets off either maladaptive apoptosis or adaptive survival (Elwakiel et al., 2024). Treatments for DKD that focus on ER stress appear to be promising (Xu et al., 2023). Genes related to unfolded protein response (UPR), associated with ER stress, were significantly upregulated in renal biopsies from DKD patients, as revealed by microarray analysis of the tubulointerstitial area (Fan et al., 2017). Ke et al. found that the ribonuclease-specific inhibitors of inositol-requiring enzyme 1α (IRE1α), an endoplasmic reticulum stress (ERS)-related molecule, and its phosphorylation were elevated after a 48-h HG induction in rat RTECs (Ruiqiong et al., 2020). A large number of ROS free radicals accumulate in cells for the activation of ERS and UPR, triggering oxidative stress response, triggering inflammation, and activating protein kinase R-like ER kinase (PERK)/C/EBP homologous protein (CHOP)/Caspase-12 and other signaling pathways, which become the basis of the pathogenesis of DKD (Ruiqiong et al., 2020).

### 3.4 Inflammation

An important part of the pathogenesis of DKD is inflammation. It has been reported that HG accelerates the occurrence and development of DKD by causing cell damage, releasing pro-inflammatory mediators such as adhesion molecules, chemokines such as tumor necrosis factor α (TNF-α) and interleukin-1β (IL-1β), which damage the kidney and promote the damage-related molecular pattern of fibrosis (Jung and Yoo, 2022). Ke et al. proposed that HG treatment elevated levels of IL-1β and IL-18 in NRK-52E cells (Ruiqiong et al., 2020). HG boosted the levels of mature IL-1β, IL-6, and TNF-α in HK-2 cells, as well as cytoplasmic nucleotide-binding oligomerization domain-like receptor 3 (NLRP3) (Xu et al., 2021).

Hence, HG treatment in RTECs leads to oxidative stress, mitochondrial damage, inflammatory responses, and apoptosis in tubular cells, ultimately contributing to the development and progression of DKD. Extensive research has identified molecular mechanisms related to inflammation in the kidney caused by diabetes, including the activation of oxidative stress (Charlton et al., 2020), Toll-like receptors (Rayego-Mateos et al., 2020), Janus kinase/signal transducer and also known as protein kinase B or PKB (AKT)/mammalian target of rapamycin (AKT-mTOR) signaling pathways, as well as the activation of nuclear factor kB (NF-kB) (Sanz et al., 2010). Additionally, epigenetic mechanisms have been identified, which result in a transcriptional reprogramming of resident renal cells (Woroniecka et al., 2011; Rayego-Mateos et al., 2023).

However, in DKD patients, glucose metabolism disorder is often accompanied by lesions of lipid metabolism, and the accumulation of lipids likewise also causes damage to renal tubular cells. Eventually, disorders of glycolipid metabolism play a vital role in renal tubulopathy in DKD together.

Lipid-mediated tubular interstitial damage caused by inflammation can occur both *in vivo* and *in vitro* via activating the scavenger receptor that binds phosphatidylserine and oxidized lipoprotein (CXCL16) pathway (Hu et al., 2018; Wang H. et al., 2017). Western blotting and immunohistochemical staining demonstrated increased CXCL16, a disintegrin and metalloproteinase family 10 (ADAM10), and a receptor for the chemokine CXCL16 (CXCR6) protein expression in db/db animals that received casein injections. In addition, mRNA (Messenger RNA) and protein expression levels of the CXCL16 pathway were elevated when HK-2 cells were activated by interleukin-1 (IL-1) (Izquierdo et al., 2012; Lin et al., 2021). These findings suggested that inflammation-induced CXCL16 pathway activation may add up to cellular fat accumulation following tubular interstitial injury. CXCL16 siRNA lowered lipid buildup and, as a result, ROS generation and extracellular matrix (ECM) excretion in HK-2 cells (Hu et al., 2018). Consistently, the research of Yang discovered that Casein-induced chronic inflammation exacerbates kidney damage in HFD-fed mice (Yang et al., 2017). Conversely, it has been confirmed that N-acetyl-β-d-glucosaminidase/growth factor-15 (NAG-1/GDF15) can block advanced glycation endproducts/the receptor for advanced glycation endproducts (AGE/RAGE)-mediated inflammatory signaling pathways in HK-2 cells and mice, hence preventing DKD (Chen et al., 2022b).

Therefore, lipid metabolism disorders damage the function of RTECs through oxidative strain, mitochondrial malfunction, and inflammation, finally leading to the death of RTECs and hypoxia.

The pathogenesis of DKD is complicated, involving the activation of various cells and signal pathways. Inflammatory response, metabolic disorders, oxidative stress, pathological changes of glomeruli and cell damage all play a crucial part in the pathogenesis of DKD, both induce the injury of mitochondria, finally leading to the dysfunction of RTECs.

## 4 Protective roles of renal tubular injury by hypoglycemic agents

### 4.1 Metformin

Metformin exerts reno-protective effects primarily through AMPK pathway activation. By upregulating AMPK signaling, metformin suppresses hypoxia-inducing factor-1α (HIF-1α), a master regulator of aerobic glycolysis implicated in hypoxia and inflammation. This inhibition attenuates high glucose- and lipid-induced oxidative stress, inflammatory responses, ER stress, mitochondrial dysfunction, apoptosis, and epithelial-mesenchymal transition (EMT), thereby mitigating RTEC injury (Song A. et al., 2021; Melnik, 2023). Furthermore, AMPK-mediated mechanisms contribute to the preservation of podocyte density, prevention of mesangial cell apoptosis, and attenuation of tubular cell degeneration in DKD (Song A. et al., 2021). Liang et al. investigated how metformin works through the cell cycle-related protein E2F translation factor 1 (E2F1) to reduce the senescence and fibrosis of renal tubular epithelial cells brought on by HG (Liang et al., 2022). According to an over 15-year follow-up study, metformin reduced diabetes-associated microvascular complications indeed, including DKD (Diabetes Prevention Program Research Group, 2015).

### 4.2 Glucagon-like peptide-1 (GLP-1)/GLP-1 receptor agonists (GLP-1RAs)

GLP-1RAs, a novel class of glucose-lowering agents, exert reno-protective effects by directly activating GLP-1 receptor signaling pathways, enhancing insulin secretion, and mitigating DKD progression (Górriz et al., 2020). Specifically, GLP-1RAs reduce ectopic lipid deposition in RTECs through multiple mechanisms, including suppression of lipogenesis, promotion of fatty acid oxidation, activation of autophagy, and improvement of insulin resistance, thereby alleviating lipid-induced cellular injury. These effects, combined with their anti-inflammatory, antioxidant, and metabolic regulatory properties, position GLP-1RAs as a promising therapeutic strategy for DKD (Su et al., 2020). Clinically, liraglutide and semaglutide are widely utilized GLP-1RAs. Experimental studies by Li et al. demonstrated that GLP-1RA administration significantly attenuates high glucose (HG)-induced RTEC injury in DKD models, potentially mediated by immunomodulatory mechanisms (Li R. et al., 2022). Research indicated that the use of GLP1-RA was associated with a lower risk of kidney outcomes. Reductions in both kidney outcomes and major adverse cardiovascular events were similar in magnitude to those reported in large cardiovascular outcome trials (Xu et al., 2022).

### 4.3 Sodium-dependent glucose transporters 2 inhibitors (SGLT2i)

SGLT2i exhibit multifaceted reno-protective effects, including glycemic control, attenuation of renal hyperfiltration, reduction of proteinuria, improvement of renal hypoxia, suppression of inflammation, blood pressure lowering, uric acid reduction, ROS scavenging, and promotion of weight loss (Cai et al., 2020; Doshi and Friedman, 2017). Mechanistically, studies by Schaub et al. demonstrate that SGLT2i ameliorates renal injury in diabetes by mitigating metabolic disturbances and inhibiting mTORC1 signaling pathways within renal tubules (Schaub et al., 2023). Randomized clinical trials have validated the additional cardiorenal benefits of SGLT2i. Patients without T2DM who have heart failure or chronic kidney disease could also benefit from SGLT2i’s cardiorenal effects (Lim et al., 2023; Laffel et al., 2023; Yang et al., 2023). Among SGLT2 inhibitors, dapagliflozin has demonstrated the most significant cardioprotective benefits, as evidenced by its ability to reduce cardiovascular mortality, heart failure hospitalization, and adverse cardiac remodeling in clinical trials.

### 4.4 Dipeptidyl peptidase-4 enzyme inhibitors (DPP4i)

DPP-4i exhibit potent anti-inflammatory properties by suppressing the expression of pro-inflammatory mediators, including TNF-α, IL-1β, monocyte chemoattractant protein-1 (MCP-1), intercellular adhesion molecule-1 (ICAM-1), transforming growth factor-β (TGF-β), and NLRP3 inflammasome activation. These effects are mediated through the restoration of AKT and ERK signaling pathways and inhibition of NF-κB activation (Gangadharan Komala et al., 2016). Additionally, DPP-4i demonstrates reno-protective effects, as evidenced by its antiproteinuric action in renal ablation models. Mechanistically, DPP-4 inhibition enhances urinary sodium excretion in distal tubules, potentially through increased circulating levels of stromal cell-derived factor-1α (SDF-1α1-67), a candidate DPP-4 substrate implicated in sodium regulation (Lovshin et al., 2017). Additionally, DPP4 urine activity that corresponds with the course of CKD has been explored. The findings demonstrate that DPP4i stopped the decline in megalin and podocin expression in CKD mice and improved both tubular and glomerular proteinuria (Benetti et al., 2021). Both DPP-4i and SGLT2i attenuate renal cell necrosis and inflammation, thereby delaying the progression of DKD. These effects are mediated through the upregulation of hypoxia-inducible factor-2α (HIF-2α) and concurrent downregulation of HIF-1α expression, which collectively improve renal oxygenation and mitigate hypoxia-induced injury (Samaan et al., 2023). It seems that DPP-4 inhibitors are beneficial for albuminuria. The Saxagliptin Assessment of Vascular Outcomes Recorded in Patients with Diabetes Mellitus (SAVOR)-Thrombolysis found that at 1 year, 2 years, and the end of treatment, saxagliptin was linked to significantly less worsening and more improvement in microalbumin levels (Bloomgarden, 2018).

### 4.5 Thiazolidinediones (TZDs)

**FIGURE 3:**
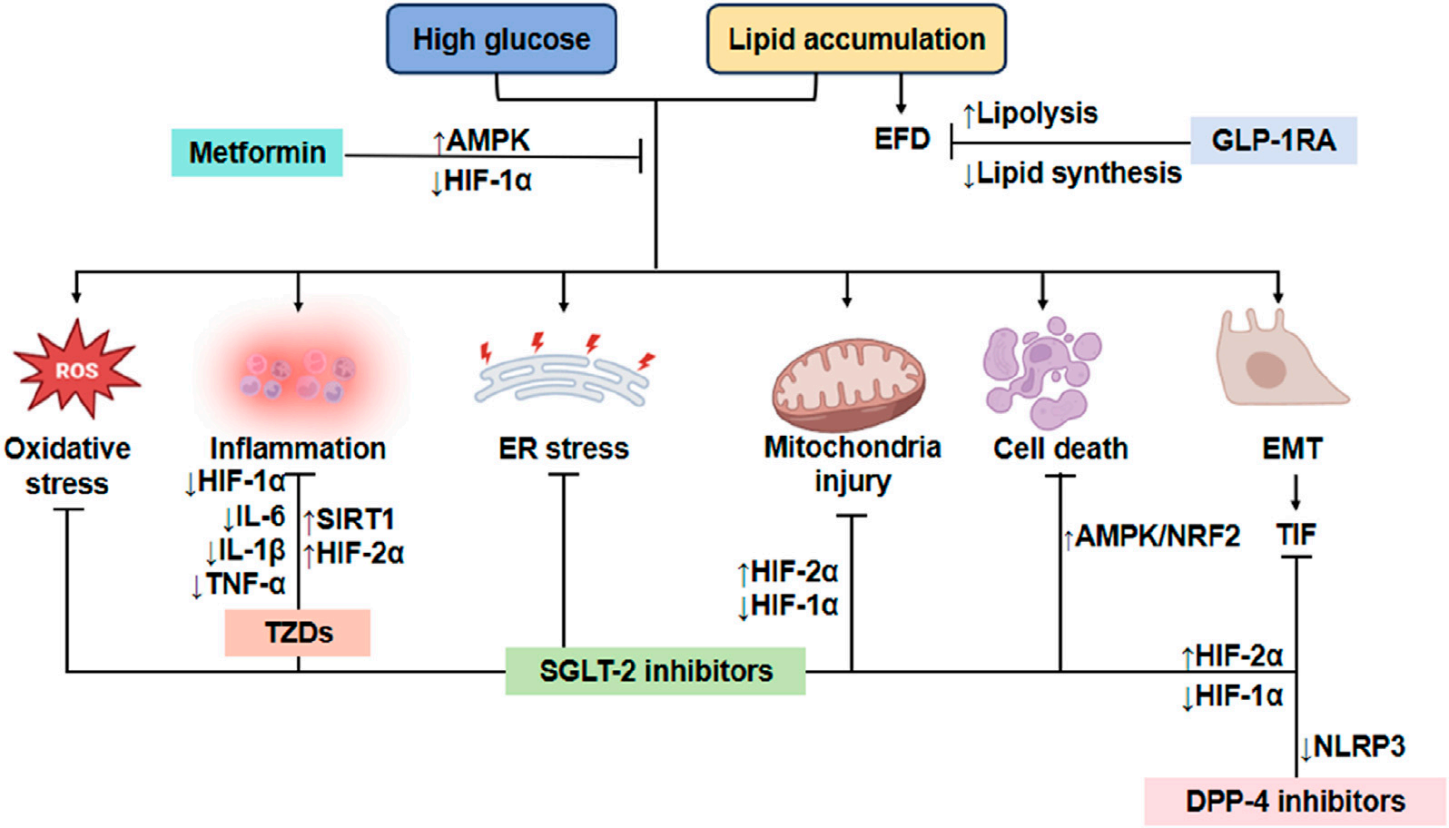
Mechanism of reducing renal tubule injury with typical hypoglycemic drugs. Metformin exerts its protective effects on RTECs by activating the AMPK pathway and suppressing HIF-1α expression, thereby mitigating oxidative stress, inflammation, ER stress, mitochondrial dysfunction, apoptosis, and EMT. Similarly, GLP-1RAs alleviate lipid toxicity in RTECs by inhibiting lipogenesis and enhancing fatty acid oxidation, which reduces EFD and associated cellular injury. Furthermore, both DPP-4i and SGLT2i attenuate renal cell necrosis and inflammation, delaying DKD progression through modulation of HIF-1α signaling. Additionally, DPP-4i suppresses NLRP3 inflammasome activation, thereby reducing tubulointerstitial fibrosis (TIF) and further preserving renal function. TZDs protects RTECs by inhibiting inflammatory cytokines. EFD, Ectopic fat deposition; ER, Endoplasmic reticulum; EMT, Epithelial-mesenchymal transition; TIF, Tubulointerstitial fibrosis; GLP-1RA, GLP-1 receptor agonists; NLRP3, nucleotide-binding oligomerization domain-like receptor 3; IL-1β, interleukin-1beta; IL-6, interleukin-6; TNF-α, tumor necrosis factor α; HIF-1α, hypoxia inducible factor-1α; HIF-2α, hypoxia inducible factor-2α; SIRT1, silent information regulator family protein 1; AMPK, adenosine 5′-monophosphate-activated protein kinase; NRF2, nuclear factor-erythroid 2-related factor 2; TIF, tubulointerstitial fibrosis; TZDs, Thiazolidinediones.

TZDs (Thiazolidinediones) are a class of oral hypoglycemic agents that improve insulin resistance by activating PPAR-γ. Commonly used clinical drugs are Pioglitazone and Rosiglitazone. Firstly, the mechanism of action is PPAR-γ activation. TZDs bind and activate nuclear receptor PPAR-γ, regulating adipocyte differentiation, lipid metabolism and insulin sensitivity. Secondly, improving insulin resistance increases glucose uptake in adipose tissue and reduces free fatty acid (FFA) release. Thirdly, inhibiting liver gluconeogenesis reduces liver sugar output. Fourthly, promoting skeletal muscle glucose utilization: enhancing insulin signaling pathways (e.g., PI3K/Akt). Lastly, the anti-inflammatory effect: Inhibit pro-inflammatory pathways such as NF-κB and reduce the release of inflammatory factors (such as TNF-α and IL-6) (Mastrototaro and Roden, 2021). The mechanisms of hypoglycemic drugs to alleviate renal tubular injury caused by disorders of glycolipid metabolism are shown in Figure 3. The clinical application of drugs to improve glycolipid metabolism and the mechanism of their influence on DKD are listed in Table 1.

**TABLE 1:** Mechanisms and existing models of typical hypoglycemic agents.

Type of drugs	Mechanism	Model
Metformin	Activate AMPK signaling pathway and inhibit NLRP3 inflammasome (Song et al., 2021b)	RTECs caused by high glucose via the E2F1 (Liang et al., 2022)
SGLT-2 inhibitors	Restrain SGLT-2 activity in proximal convoluted tubules (Doshi and Friedman, 2017)	The diabetic model (KK-Ay mice) was administrated with englipzin (Liu et al., 2020)
GLP-1 receptor agonist	Modulate cAMP/PKA signaling and inhibit RAAS (Górriz et al., 2020)	GLP-1RA treatment mice and HG-induced RTECs (Li et al., 2022b; Kawanami and Takashi, 2020; Giugliano et al., 2019)
DPP-4 enzyme inhibitors	Inhibits GLP-1 degradation and prolongs insulin action (Gangadharan Komala et al., 2016)	Male Wistar rats were treated with sitagliptin after nephrectomy (Benetti et al., 2021)

(Note: AMPK, adenosine 5′-monophosphate-activated protein kinase; NLRP3, nucleotide-binding oligomerization domain-like receptor 3; RTECs, renal tubular epithelial cells; E2F1, cell cycle-related protein E2F translation factor 1; SGLT2i, Sodium-dependent glucose transporters 2 inhibitors; RNA, ribonucleic acid; cAMP/PKA, Cyclic Adenosine Monophosphate/Protein Kinase A Signaling Pathway; RAAS, Renin-Angiotensin-Aldosterone System; GLP-1, Glucagon-like peptide-1; GLP-1Ras, GLP-1, receptor agonists; DPP4i, Dipeptidyl peptidase-4, enzyme inhibitors.).

## 5 Protective roles of renal tubular injury by antihyperlipidemic agents

### 5.1 Statins

Statins are widely regarded as the first-line pharmacological intervention for atherosclerotic cardiovascular diseases due to their potent lipid-lowering and pleiotropic effects. Commonly prescribed agents include Atorvastatin and Rosuvastatin, which have demonstrated efficacy in reducing LDL cholesterol levels and stabilizing atherosclerotic plaques. The enzyme that restricts the rate of cholesterol production is termed a reductase (Ali et al., 2021). Because of their part in reducing plasma fat, statins have numerous anti-atherosclerotic actions. Early animal research has indicated that statin therapy may offer renoprotection in chronic renal disease patients, surpassing the benefits of strict blood pressure management and angiotensin II antagonist use (US Preventive Services Task Force et al., 2022). One of the main clinical issues with nephrotoxicity has been suggested to be the buildup of aminoglycoside (AG) drugs in the renal proximal tubes. This process can be modulated by the production of 3-hydroxy-3-methylglutaryl-CoA (HMG-CoA) (Antoine et al., 2010). Antoine investigated the theory that statins will lessen AG renal proximal tubule buildup and toxicity because they decrease HMG-CoA (Antoine et al., 2010). The study found that statins’ regulation of the relevant pathway provides a workable therapeutic strategy to halt AG-induced nephrotoxicity (Sattar, 2023). Furthermore, compared to non-treated obese rats, animal models of obesity demonstrate that all statins reduced lipid accumulation in the proximal tubules, improved glomerular hypertrophy, raised nephrin expression, and decreased desmin expression (Gotoh et al., 2013). The information that is now available supports the cardiovascular advantages of statins in individuals with chronic renal disease, and starting a statin was strongly linked to a lower risk of death from all causes (Barayev et al., 2023; Navaneethan et al., 2006). Further studies have demonstrated that fluvastatin effectively suppresses NF-κB activation in renal tubular epithelial cells (RTECs) under hyperglycemic conditions, both *in vivo* and *in vitro* (Gao et al., 2013).

### 5.2 Fibrates

Fibrates, a class of PPAR agonists, are clinically effective for lipid profile modification. Commonly prescribed agents include fenofibrate and gemfibrozil, which primarily target triglyceride reduction and HDL cholesterol elevation (Kim and Kim, 2020). PPAR is a crucial regulator of thrombogenesis, lipid metabolism, and inflammation (Fardoun et al., 2017). The activation of PPAR by fibrates results in a wide range of anti-atherogenic actions. Fibrates also have antioxidant, anti-inflammatory, and anti-thrombotic properties. Owing to their potent suppression of the nuclear factor kappa B (NF-kB) signaling cascade, they reduce the inflammatory process (Zivkovic et al., 2023). Fenofibrate mitigated renal fibrosis and prevented cell death by increasing PPARα in rat models of DKD (Cheng et al., 2016). Nevertheless, in a prior study using a murine acute tubular injury model known as free fatty acid (FFA) -overload nephropathy, high-dose PPAR agonist therapy (0.5% clofibrate diet) was reported to exacerbate tubular damage due to increased serum buildup of clofibrate metabolites due to impaired renal clearance (Takahashi et al., 2011). Pretreatment with low-dose clofibrate protects against acute tubular damage while preventing the accumulation of its metabolites (Takahashi et al., 2011). This delicate balance appears to be efficiently influenced by drug dosage. Patients with CKD who utilize fibrates, particularly fenofibrate, had a lower risk of major adverse cardiovascular events (MACE) both recently and currently. Nevertheless, additional research involving diverse populations is necessary to validate the applicability of these conclusions (Goto et al., 2024).

### 5.3 Bile acid sequestrants (BAS)

Bile acid sequestrants (e.g., colesevelam, colestipol, and cholestyramine) exert their therapeutic effects by binding to bile acids within the intestinal lumen, primarily in the duodenum and jejunum. This binding prevents the reabsorption of bile acids via the apical sodium-dependent bile acid transporter (ASBT) in the terminal ileum, thereby interrupting enterohepatic recirculation. The resultant depletion of hepatic bile acid pools upregulates cholesterol 7α-hydroxylase (CYP7A1), driving hepatic conversion of cholesterol to bile acids and ultimately reducing serum LDL-cholesterol levels (Zivkovic et al., 2023). In a recent study in 2021, Tamara R. Castañeda’s team created SAR442357, a unique polymeric sequestrant with enhanced phosphate and bile acid sequestration properties (Castañeda et al., 2021). In a single treatment drug, the novel polymeric sequestrant provided integrated pharmacological effects such as glucose regulation, cholesterol reduction, and protection of DKD progression (Castañeda et al., 2021). Studies on humans and animals with diabetes that demonstrate abnormal bile acid metabolism point to a connection between bile acids and glucose regulation (Staels and Kuipers, 2007). In lipid-lowering trials, bile acid sequestrants have been demonstrated to reduce levels of glycosylated hemoglobin and plasma glucose, indicating the potential use of these drugs as T2DM treatment (Staels and Kuipers, 2007).

### 5.4 Niacin

Niacin has been used to change plasma lipid levels for about 60 years. It is uncertain what exact mechanism underlies these effects. It mostly results in a drop in TG levels. Niacin’s capacity to stop adipose tissue’s production of nonesterified fatty acids (NEFAs) may be the cause of the drop in plasma TG (Marston et al., 2019). This results in a drop in NEFAs plasma concentrations, decreased NEFAs hepatic uptake, and decreased triglyceride synthesis in the liver (Katsiki et al., 2018). Copper-nicotinate complex (CNC) has antioxidant qualities that protect the kidneys by scavenging free radicals that the body produces. CNC reduces fibrosis, degenerative alterations, and necrotic changes in the renal tubules in the rat model of kidney damage caused by TG through its antioxidant action (Medhat Hegazy et al., 2020). Drugs that lower TG, including sustained-release niacin tablets, have been linked in randomized controlled studies to a decreased risk of major vascular events (Marston et al., 2019).

### 5.5 Proprotein convertase subtilisin/kexin type 9 (PCSK9) inhibitors

PCSK9 is a brand-new target for lipid-lowering treatment. The drugs that are widely used in our country at present include Aliciumab and Ilozumab. Recently, PCSK9 inhibitors have been touted as effective treatments for reducing LDL-C. Hepatic LDL receptors (LDL-R) are degraded due to the protein PCSK9. Evolocumab and alirocumab, two monoclonal antibodies, effectively reduce PCSK9 activity (Writing et al., 2022). LDL-C levels almost always rise in nephrotic syndrome patients. Furthermore, elevated LDL-C may harm mesangial, tubular, and podocyte renal cell types. Liver cells overexpress PCSK9, which increases the degradation of LDL-R in nephrotic syndrome (Busuioc et al., 2020). In brief, PCSK9 therapeutic regulation using antibody therapy may be a viable option. These combined safety and effectiveness data demonstrate that PCSK9 is a safe, well-tolerated, and effective treatment to lower LDL-C in adults with heterozygous familial hypercholesterolemia, atherosclerotic cardiovascular disease (ASCVD), or ASCVD risk equivalents when given twice a year in addition to maximally tolerated statin therapy with or without other LDL-C lowering agents (Rosenson et al., 2018; Wright et al., 2021; Wang et al., 2022).

### 5.6 Bempedoic acid

An innovative oral lipid-lowering drug called bempedoic acid became available in Europe in November 2020. Bempedoic acid, like statins, decreases cholesterol production. For people currently taking statins, it can reduce LDL cholesterol even though it blocks the same mechanism (Rubino et al., 2021). In individuals with mild, moderate, or severe renal impairment, the estimated area under the concentration-time curve exposure following bempedoic acid medication was higher than in those with normal renal function (Amore et al., 2022). Bempedoic acid prevented vascular remodeling and preserved vascular integrity by obstructing the ERK/transforming growth factor-fibrotic pathway (Ahmed et al., 2023). This may predict also make significant effect in capillary around renal tubules (Ahmed et al., 2023). A clinical trial showed that bempedoic acid medication was linked to a decreased risk of significant adverse cardiovascular events (cardiac mortality, nonfatal myocardial infarction, nonfatal stroke, or coronary revascularization) in individuals who were intolerant to statins (Nissen et al., 2023).

### 5.7 Omega-3 fatty acids

Omega-3 fatty acids, high-purity fish oil preparation (ethyl eicosapentaenoate) reduce blood TG concentrations via reducing TG synthesis, TG integration into Very Low-Density Lipoprotein (VLDL), TG secretion, and TG clearance from VLDL particles. Specifically, it lowers sterol regulatory element-binding protein-1c expression to make cholesterol levels decrease (Marston et al., 2019; Backes et al., 2016). Palmitic acid induces endoplasmic reticulum (ER) stress, leading to both necrotic and apoptotic cell death in renal proximal tubular cell lines (Katsoulieris et al., 2009). In contrast, unsaturated fatty acids may lessen the lipotoxicity linked to saturated fatty acids by inhibiting ER stress and subsequently reducing the synthesis of C/EBP analogous proteins and apoptotic renal cell death (Katsoulieris et al., 2009). Jeong et al. suggest that docosahexaenoic acid (DHA), one of the most important omega-3 fatty acids, can inhibit inflammatory factors and oxidative stress (Jeong et al., 2023). The benefits of omega-3 fatty acids, especially those obtained from marine sources, seem to outweigh their tendency to decrease cholesterol in randomized controlled trials (Marston et al., 2019).

### 5.8 Ezetimibe

**FIGURE 4:**
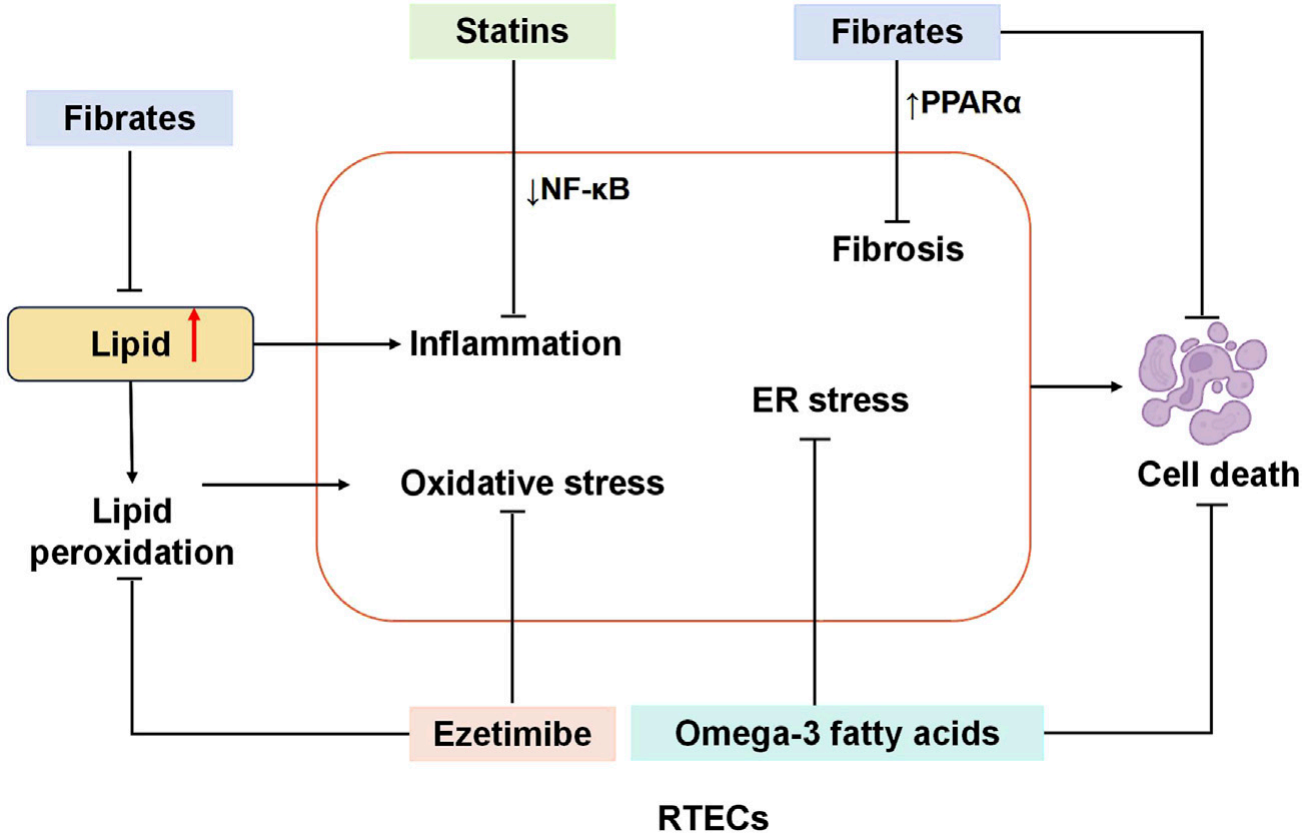
Mechanism of reducing renal tubule injury with typical lipid-lowering drugs. Statins attenuate RTEC injury by downregulating NF-κB signaling, thereby suppressing inflammatory responses. Fibrates exert reno-protective effects through dual mechanisms: directly reducing TG synthesis and upregulating PPARα to counteract renal fibrosis and apoptosis. Ezetimibe mitigates RTEC damage by inhibiting lipid peroxidation and enhancing renal antioxidant enzyme activity. Similarly, omega-3 fatty acids protect RTECs by alleviating ER stress and reducing apoptotic cell death. RTECs, renal tubular epithelial cells; NF-κB, nuclear factor κB; PPAR-α, peroxisome proliferators-activated receptors; ER, Endoplasmic reticulum.

The cholesterol absorption inhibitor ezetimibe inhibits the hepatocytes and gastrointestinal tract’s Niemann-Pick C1 like 1 protein (NPC1L1). LDL receptor activation lowers plasma LDL-cholesterol levels by blocking the intestinal absorption of cholesterol. Recent research indicated that ezetimibe (EZ) remarkably increased the microalbumin and characteristics of renal histopathology that are protected. In diabetic mice, EZ demonstrates reno-protective and antidiabetic effects by reducing lipid peroxidation and enhancing the activity of kidney antioxidant enzymes (Nabi et al., 2023; Shen et al., 2023). A randomized controlled trial revealed that for the 3-year composite outcomes, moderate-intensity statin combination therapy with ezetimibe was not inferior to high-intensity statin monotherapy among patients with ASCVD. This was due to a higher proportion of patients having LDL cholesterol concentrations of less than 70 mg/dL and a lower incidence of drug discontinuation or dose reduction caused by intolerance (Kim et al., 2022). The mechanisms of lipid-lowering drugs to alleviate renal tubular injury caused by disorders of lipid metabolism are shown in Figure 4. Lipid-lowering drugs and their mechanisms of influence on DKD are summarized in Table 2.

**TABLE 2:** Main efficacy and known models of lipid-lowering agents.

Type of drugs	Mechanism	Mainly effect on lipid parameters LDL-c HDL-c TG			Model
Statins	HMG-CoA reductase inhibitors prevent the production of cholesterol (Laakso and Fernandes Silva, 2023)	✓			Fluvastatin reduces NF-κB activation induced by RTECs hyperglycemia *in vivo* and *in vitro* (Gao et al., 2013)
Fibrates	Activate PPAR-α and promote TG hydrolysis (Fardoun et al., 2017)			✓	Fenofibrate alleviates renal fibrosis by increasing PPARα in a DKD rat model (Cheng et al., 2016)
Bile acid sequestrants	Prevent intestinal absorption of cholesterol (Zivkovic et al., 2023)	✓			SAR442357 for ZSF1 rats (Castañeda et al., 2021)
Nicotinic acid	Reduce liver synthesis and secretion of VLDL, increase HDL.			✓	Male Wistar rats were treated with CNC (Medhat Hegazy et al., 2020)
PCSK9	Prevent PCSK and LDL-R degradation	✓			Rats with puromycin-induced DKD treated with PCSK9 (Busuioc et al., 2020; Khan et al., 2022)
Bempedoic acid	Decreases cholesterol production	✓			Male Sprague-Dawley rats were treated with benzopolyacid (Amore et al., 2022; Ahmed et al., 2023)
Omega-3 fatty acids	Reduces TG concentrations			✓	Omega-3 fatty acids can improve the transport function of RTECs in AKI mouse models (Rund et al., 2020)
Cholesterol absorption inhibitor	Inhibition of cholesterol absorption in the intestine	✓			STZ-induced diabetic rats treated with Ezetimibe (Mori and Hirano, 2012)

(Note: HMG-CoA, 3-hydroxy-3-methylglutaryl-CoA; PPAR-α, peroxisome proliferators-activated receptors; TG, triglycerides; ZSF1, Eight-week-old male Zucker fatty/spontaneously hypertensive heart failure F1 hybrid rats; VLDL, Very Low-Density Lipoprotein; HDL, High-Density Lipoprotein; CNC, Copper-nicotinate complex; PCSK9, Proprotein convertase subtilisin/kexin type 9; LDL-R, Low-Density Lipoprotein Recepter; DKD, diabetic kidney disease; STZ, streptozotocin).

## 6 Limitations

This review has several inherent limitations in its comprehensive analysis of renal tubular injury mechanisms. First, while we emphasized triglyceride-induced dyslipidemia as a key contributor to tubular damage, the potential role of cholesterol-mediated lipid metabolism disorders remains underexplored. Second, our focus on clinically established therapeutic agents targeting renal tubules, though mechanistically relevant, reflects a limited coverage of current glucose- and lipid-lowering pharmacotherapies. Notably, agents with weaker direct tubular effects were not systematically evaluated.

Additionally, due to scope constraints, we could not fully delineate the pathophysiological interplay between specific lipid subtypes and tubular dysfunction. Lastly, while pharmacological interventions were prioritized, non-pharmacological strategies, particularly dietary interventions such as glycemic index modulation and saturated fat restriction, were not addressed despite their proven efficacy in population-specific management of metabolic disorders.

## 7 Conclusion

**FIGURE 5:**
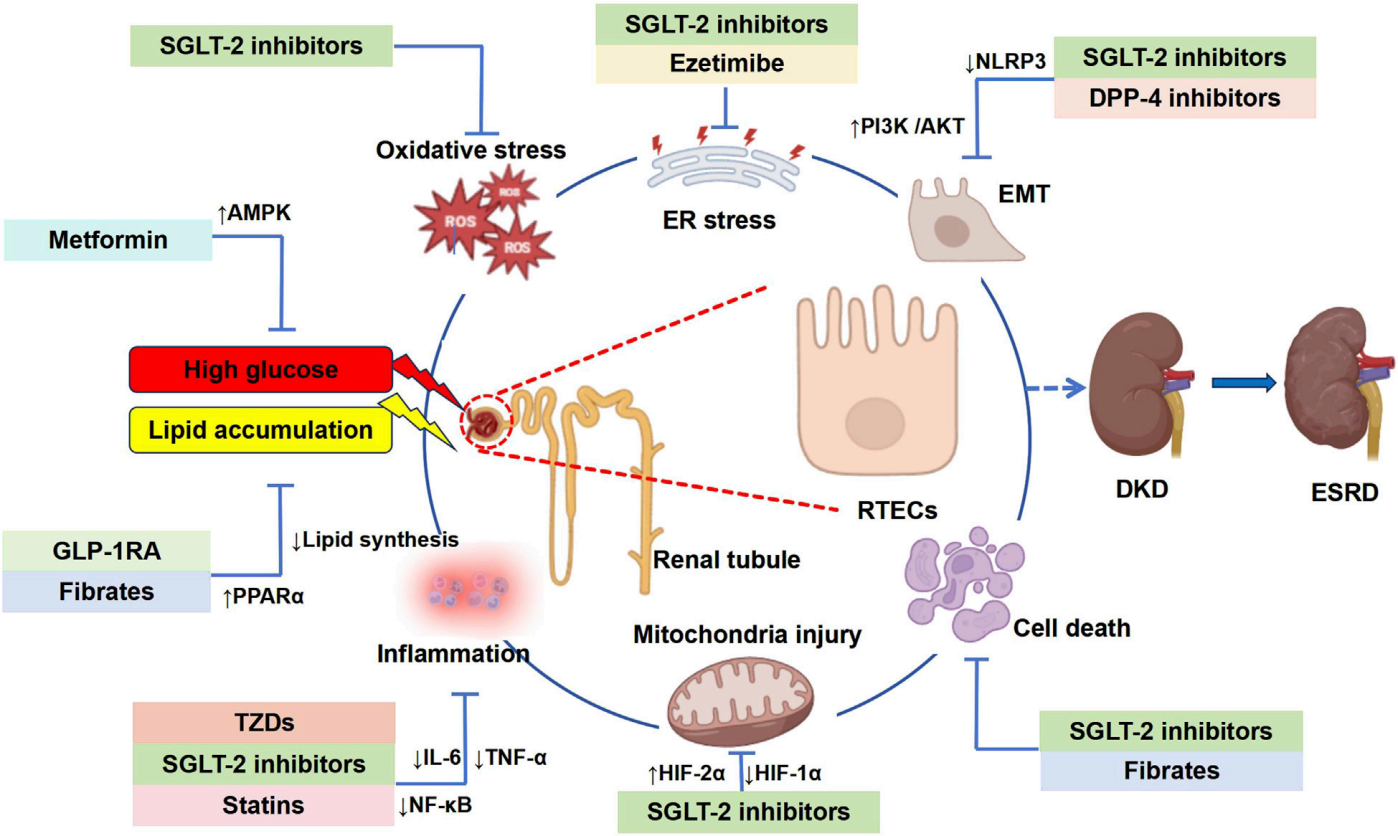
Graphic summary: Mechanisms of glycolipid metabolism disorders leading to DKD and clinical drugs. This figure provides a comprehensive overview of the hypoglycemic and lipid-lowering agents discussed in this review, highlighting their mechanisms in mitigating DKD-associated tubular injury. RTECs, renal tubular epithelial cells; DKD, diabetic kidney disease; ESRD, end-stage renal disease; EFD, ectopic fat deposition; ER, endoplasmic reticulum; ROS, reactive oxygen species; NLRP3, nucleotide-binding oligomerization domain-like receptor 3; NF-κB, nuclear factor κB; IL-6, interleukin-6; TNF-α, tumor necrosis factor α; EMT, epithelial-mesenchymal transition; HIF-1α, hypoxia inducible factor-1α; HIF-2α, hypoxia inducible factor-2α; AMPK, adenosine 5′-monophosphate-activated protein kinase; PPAR-α, peroxisome proliferators-activated receptors; PI3K/AKT, phosphatidylinositide 3-kinases/protein kinase B or PKB.

Renal tubule damage is thought to occur in the early stage of DKD and plays an important role in the occurrence and development of DKD. In this review, we summarize the mechanism of glycolipid metabolism disorders in DKD on RTECs injury. The mitochondrial damage caused by glycolipid metabolism disorder plays a key role in RTECs injury. At the same time, the possible mechanism of corresponding therapeutic drugs in the application of DKD was discussed (summarized in Figure 5). However, further research is still needed to identify the mechanisms of drugs that effectively inhibit and treat DKD, as well as drugs that target RTECs damage. With the emergence of the “tubulocentric view of DKD”, the role of metabolic changes in RTECs should be paid considerable attention.
